# Atypical Presentation of Primary Hyperparathyroidism as Recurrent Pancreatitis: A Case Report With a Review of the Literature

**DOI:** 10.7759/cureus.41140

**Published:** 2023-06-29

**Authors:** Gaurav Mehta, Vaishnavi M Rathod, Tejasvi Patel, Dipak Solanki

**Affiliations:** 1 Department of General Medicine, Sir Sayajirao General (SSG) Hospital, Medical College Baroda, Vadodara, IND

**Keywords:** parathyroidectomy, parathyroid hormone (pth), hypercalcemia, pancreatitis, primary hyperparathyroidism (phpt)

## Abstract

The majority of the patients with primary hyperparathyroidism (PHPT) are asymptomatic. The most common organ systems involved in PHPT are the kidneys and the skeletal system. In rare instances, acute or chronic pancreatitis may be presenting feature in PHPT patients. The association between these both diseases is still the topic of debate. Here, we put forth a case of a 52-year-old female with three episodes of pancreatitis in the last six months who was diagnosed with PHPT during the fourth episode of pancreatitis based on raised serum amylase and serum lipase levels along with ultrasonography (USG) findings of the abdomen. Pancreatitis in the absence of additional risk factors such as gallstones and alcohol abuse along with raised parathyroid hormone (PTH), hypercalcemia and osteolytic bone lesions led us towards the diagnosis of PHPT. On radio imaging such as MRI and CT scans of the neck, parathyroid adenoma was found in the posterior aspect of the right lobe of the thyroid. She was treated with parathyroidectomy. Serum calcium and PTH levels normalised postoperatively. As can be seen from our case, recurrent pancreatitis with hypercalcaemia should be evaluated for PHPT.

## Introduction

After diabetes and thyroid disorders, primary hyperparathyroidism (PHPT) is the third most common endocrine disorder with an incidence in the general population of 28 cases in 100,000. The common age of presentation is between 50 to 60 years with female preponderance. The most common cause of PHPT is solitary parathyroid adenoma [1]. The parathyroid gland plays a major role in maintaining normal calcium levels in the blood. In PHPT, excessive amounts of parathyroid hormone (PTH) are secreted by parathyroid glands resulting in hypercalcemia. Predominant manifestations of PHPT are stones (renal stone disease), bone issues (osteitis fibrosa cystica, fracture), abdominal groans (gallstone disease, pancreatitis, acid peptic disease), psychiatric moans (mood disorders), and fatigue overtones (myalgia and myopathy) [2]. In a few cases, pancreatitis has been reported in association with PHPT. Hypercalcemia is a rare metabolic cause of pancreatitis [3]. The association between PHPT and pancreatitis is still a topic of debate. According to some studies, hypercalcemia is associated with different types of pancreatitis including acute, sub-acute and chronic pancreatitis [4]. Prevention of further episodes of pancreatitis after parathyroidectomy supports this association. Here we report a case showing the rare association between PHPT and pancreatitis.

## Case presentation

A 52-year-old postmenopausal woman was brought to our emergency department with the complaint of epigastric pain for a week prior to presentation. The pain was intermittent, severe in intensity, radiating to the back, relieved on stooping forwards, and associated with nausea and vomiting. The patient denied any history of alcohol abuse or medication use and there was no family history of hyperlipidemia or pancreatitis. Her past medical history was significant for three similar episodes of severe abdominal pain with vomiting in the last six months, which were managed conservatively. She had a significant history of fracture of the right tibia after trivial trauma three months ago, which was treated with plating and not investigated further. At the time of presentation, she did not have any history of bone pains.

On examination, she had signs of dehydration (dry mucous membranes and raised capillary refill time) and postural hypotension with tachycardia. While examining the abdomen, tenderness was elicited in the epigastric region on palpation without any rebound tenderness, rigidity, or guarding. The central nervous system, cardiovascular system, and respiratory system examinations were unremarkable. Due to the presence of typical epigastric pain with signs and symptoms of pancreatitis, we started investigating the same. Blood investigations showed raised serum lipase and raised amylase levels (Table 1). Ultrasonography (USG) abdomen and pelvis showed a bulky pancreas with altered echotexture and peripancreatic fat stranding, as well as tiny non-obstructive bilateral renal calculi. There was no evidence of gallstone disease. On the basis of clinical features, significant past history, examination findings, and investigations, a provisional diagnosis of recurrent pancreatitis was made. Further investigations were carried out to know the aetiology of pancreatitis. Elevations in serum calcium and ionised calcium were found (Table 2). Serum albumin, lipid profile, liver function tests, kidney function tests, C-reactive protein (CRP) and haematological tests were normal (Table 1). Pancreatitis was managed by keeping the patient nil per oral with fluid resuscitation, electrolyte repletion and analgesics.

**Table 1:** Laboratory Parameters

Investigations	Patient’s Value	Normal Range
Hemoglobin	11.3	11-15 gm/dl
Total count	9800	4000-10000 /cmm
Platelets	249000	150000-410000 /cmm
Urea	14	14-40 mg/dl
Creatinine	1.07	0.6-1.2 mg/dl
Sodium	138	135-145 mEq/l
Potassium	4.7	3.5-4.1 mEq/l
Total Bilirubin	0.6	0.1-1.2 mg/dl
Direct Bilirubin	0.2	0-0.4 mg/dl
Alanine Transaminase	25	0-40 IU/L
Aspartate Transaminase	34	0-37 IU/L
Alkaline Phosphate	139	28-111 U/L
Total protein	6.60	6-8 gm/dl
Albumin	4.1	3.2-5 gm/dl
Lipase	264	0-60 IU/L
Amylase	293	0-90 IU/L
Total Cholesterol	138	150-220 mg/dl
Triglycerides	108	0-150 mg/dl
Low Density lipoprotein	77	60-130 mg/dl
High Density lipoprotein	39	40-70 mg/dl
C-Reactive Protein	2.0	0-6 mg/L
Erythrocyte Sedimentation Rate	18	0-19 mm/hour

**Table 2:** Pre-Operative serial serum Calcium, ionised Calcium, intact Parathyroid Hormone (iPTH) level and Phosphorus

Investigations	On Admission	On Day 3	Normal Value
Total Calcium	14.3	12.32	8.5-10.5 mg/dl
Ionised Calcium	1.78	1.67	1.12-1.32 mmol/L
Intact Parathyroid Hormone (PTH)	1559	1642	15-68.3 pg/mL
Phosphorus	2.01	2.10	2.5-4.5 mg/dL

After the episode of acute pancreatitis subsided, serum biochemical markers were repeated. Serum calcium was still high and phosphate levels were reduced (Table 2). Further investigations were arranged to find out the underlying cause of hypercalcaemia. Serum 25-hydroxyvitamin D was normal; however, the serum intact PTH (iPTH) levels were raised (Tables 2-3). Electrocardiogram (ECG) findings, two-dimensional echocardiography (2D ECHO), serum prolactin and thyroid profile were also normal (Table 3). Based on the discovery of these biochemical parameters, a diagnosis of PHPT was made.

**Table 3:** Other relevant biochemical investigations

Investigations	Patient’s Value	Normal Range
25-hydroxyvitamin D	14.34	20-40 ng/ml
Prolactin	17	<25 ng/ml
Thyroid Stimulating Hormone (TSH)	2.01	0.27-4.2 microIU/L
Free T3	3.17	2-4.4 pg/ml
Free T4	0.98	0.93-1.7 ng/dL

**Figure 1:**
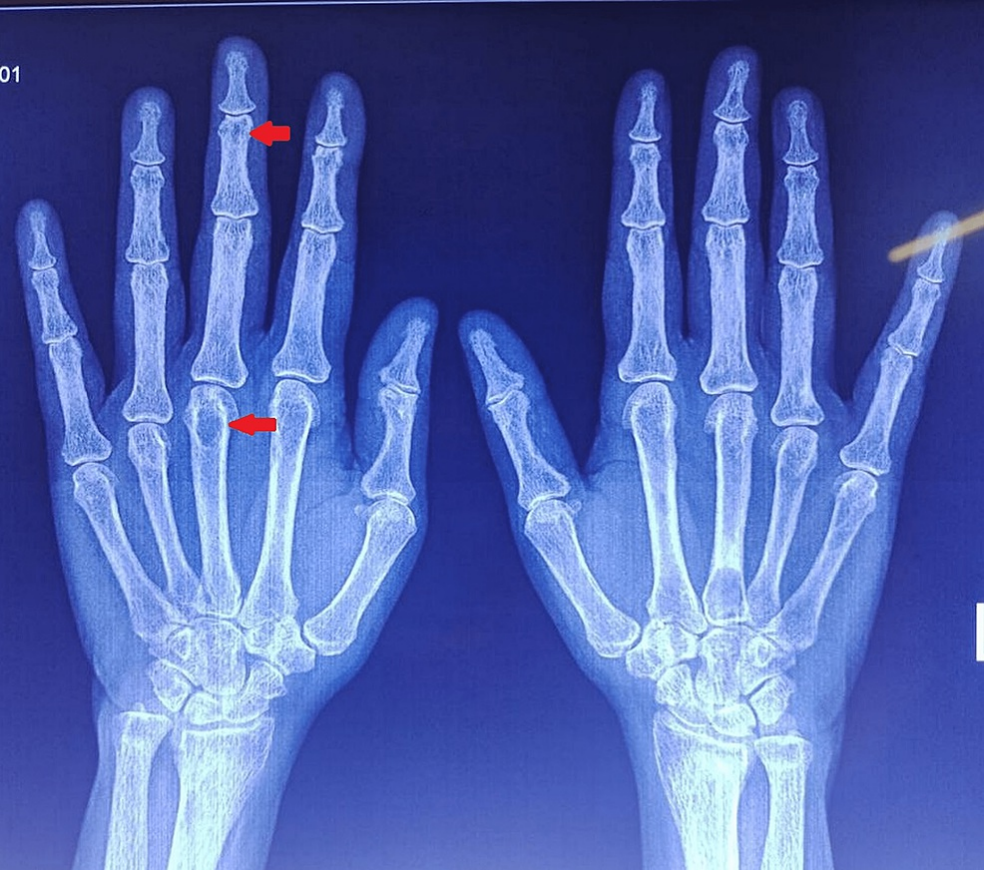
An X-ray of the hand (antero-posterior view) Lytic bone lesions are seen (red arrow) in the head and base of the metacarpals.

**Figure 2:**
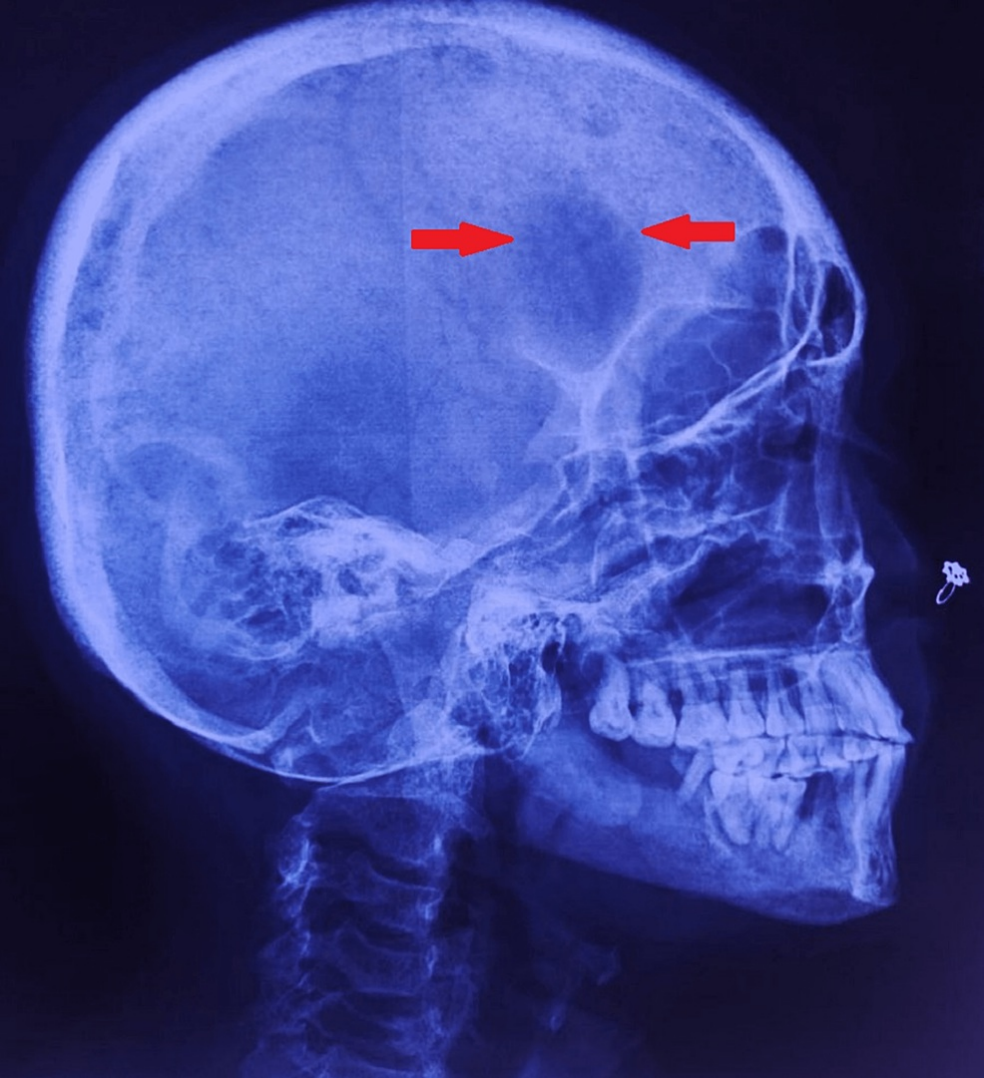
X-ray of the skull (lateral view) Multiple tiny well-defined lucencies are seen (red arrow) in the calvaria - "salt and pepper skull".

**Figure 3:**
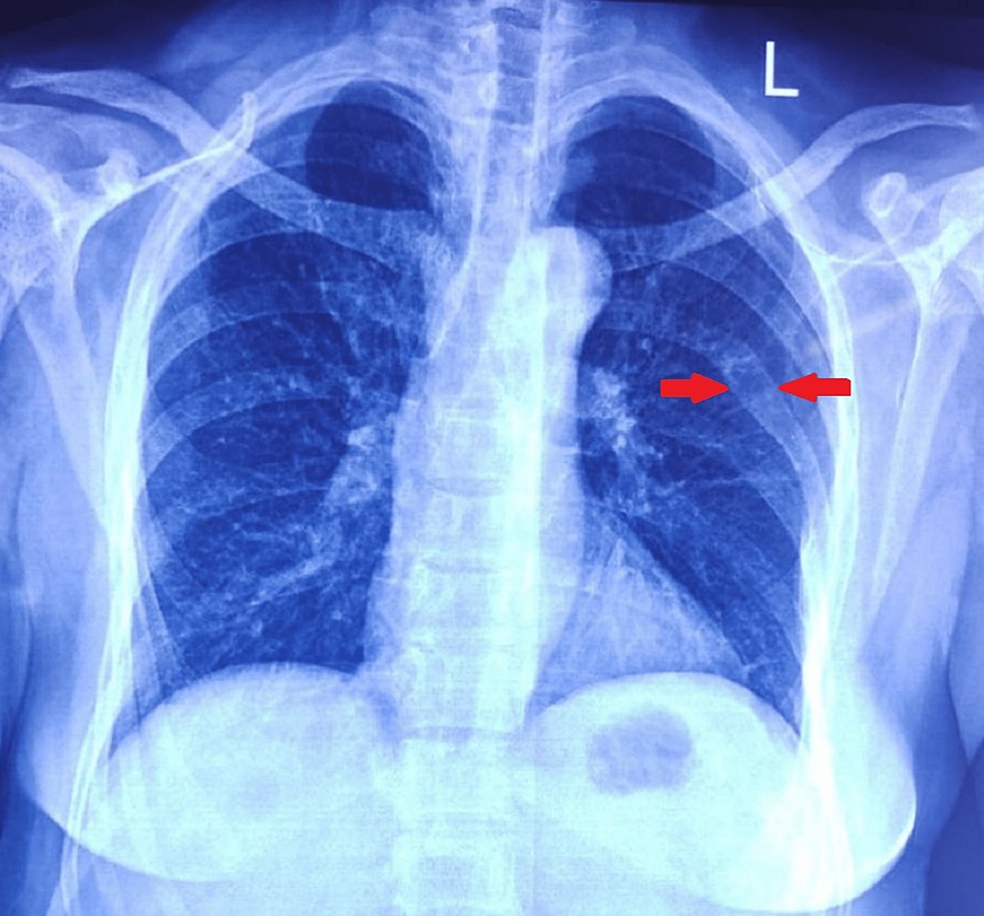
X-ray of the chest (postero-anterior view) Lytic lesions are seen (red arrow) involving the posterior aspect of the left sixth rib.

**Figure 4:**
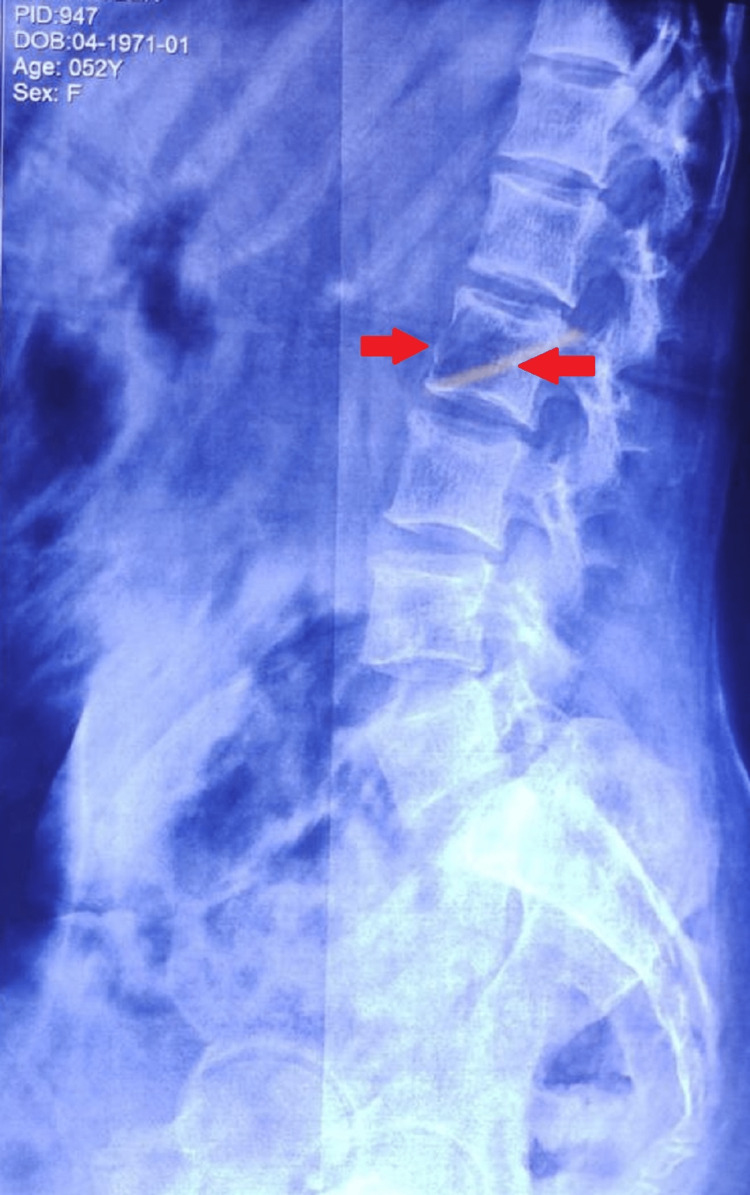
X-ray of the umbo sacral spine (lateral view) Lytic lesions are seen (red arrow) in the second lumbar vertebrae.

**Figure 5:**
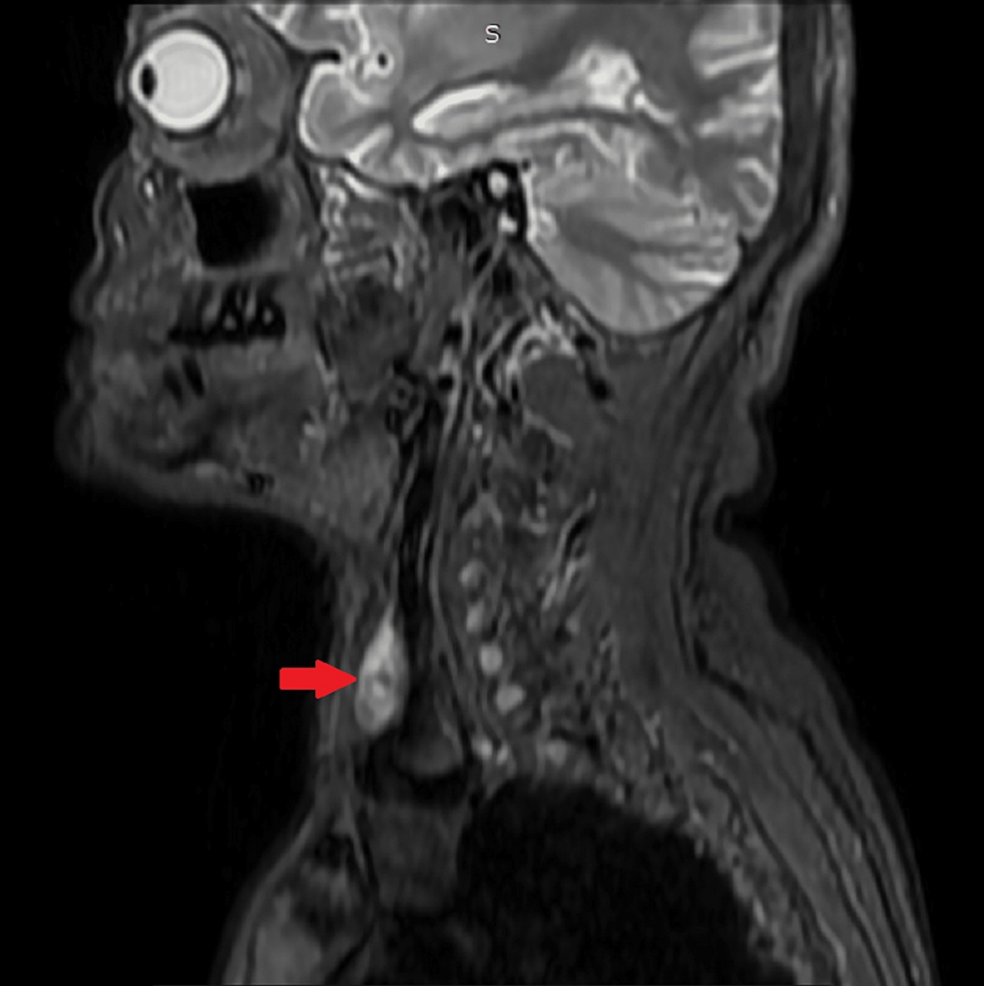
MRI of the neck in sagittal view (STIR) A focal, well-defined, heterogeneously hyperintense lesion can be seen (red arrow), consistent with parathyroid adenoma. STIR: short inversion time inversion recovery

The patient denied any history of bone pain, neuropsychiatric symptoms, or muscle weakness. Upper gastrointestinal (UGI) endoscopy ruled out gastrinoma (neuroendocrine tumour) and the possibility of multiple endocrine neoplasia type 1 (MEN1) syndrome. USG and MRI of the neck (both plain and with contrast) helped to confirm the diagnosis of right inferior solitary parathyroid adenoma with multiple osteolytic lesions in the hand, skull, rib and spine (Table 4) (Figures 1-5).

**Table 4:** Radiological investigations, suggestive of pancreatitis and osteolytic lesions with parathyroid adenoma

Investigations	Remarks
X-Ray	Hand (Antero-posterior AP view) - Lytic bone lesions were noted in the head and base of the metacarpals (Figure 1). Skull (Lateral view) - multiple tiny well-defined lucencies were noted in calvaria - " salt and pepper skull" (Figure 2). Chest (Postero-Anterior PA view)- Lytic lesions were noted involving the posterior aspect of the left sixth rib (Figure 3). Lumbo sacral spine (lateral view) - Lytic lesions were noted in the L2 vertebrae (Figure 4).
USG	Neck - well-defined, densely hypoechoic lesion on the right side of the neck along the posteroinferior aspect of the right lobe of the thyroid along with internal vascularity within (28×10mm), likely to represent parathyroid adenoma. Abdomen and pelvis - bulky pancreas with altered echotexture and peripancreatic fat stranding was noted. Multiple calculi of 4mm size in the mid pole and of a 3mm size near the junction of the upper and mid pole of the left kidney were noted. A calculus of 4mm size was noted near the junction of the mid and lower pole of the right kidney.
MRI	Neck and thorax - Focal well-defined abnormal signal intensity lesion, heterogeneously hyperintense on Short Inversion Time Inversion Recovery (STIR), intermediate on T1 images, moderate heterogeneous enhancement was seen on the right side, posterior to the right lobe of the thyroid gland, measures approximately 3.2×1.0×2.0 cm in size, consistent with parathyroid adenoma (Figure 5).

**Figure 6:**
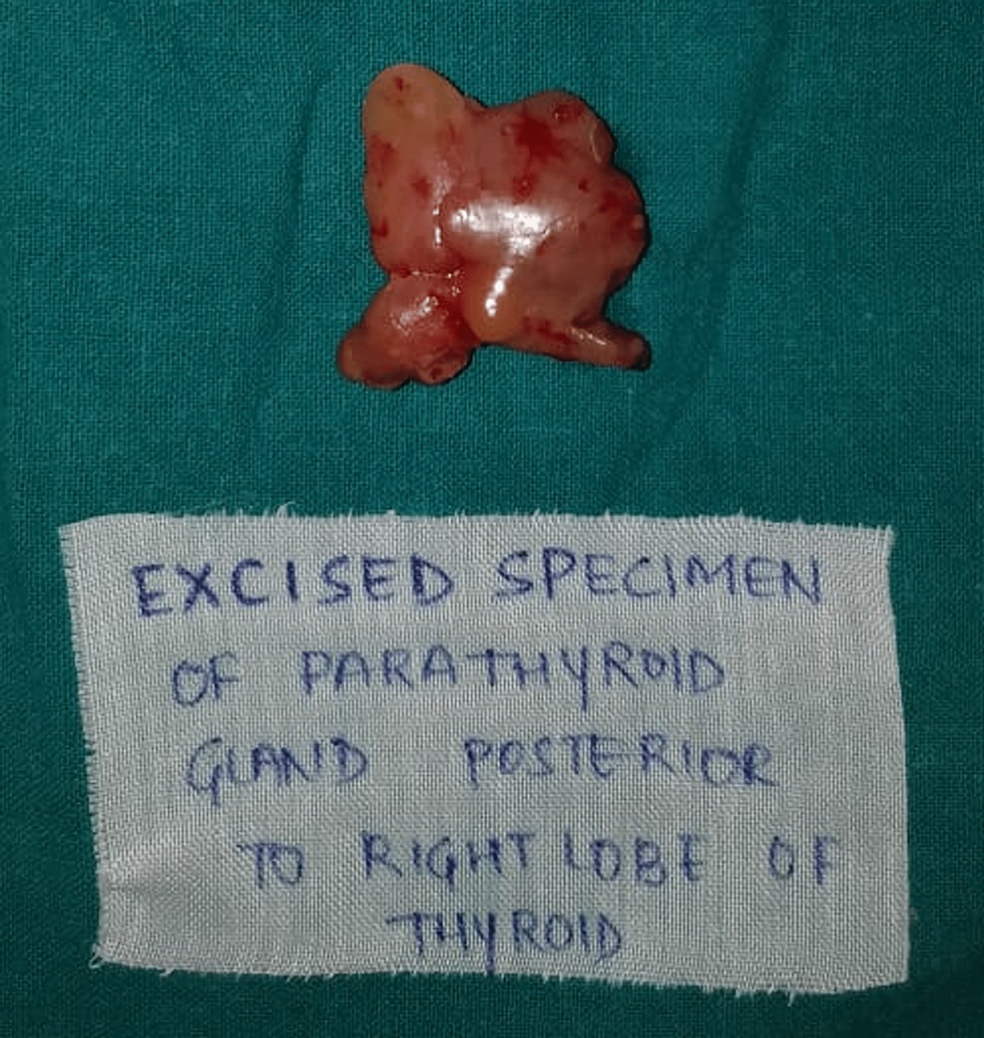
Specimen of 3x2.5x1 cm sized excised adenoma of the parathyroid gland

**Figure 7:**
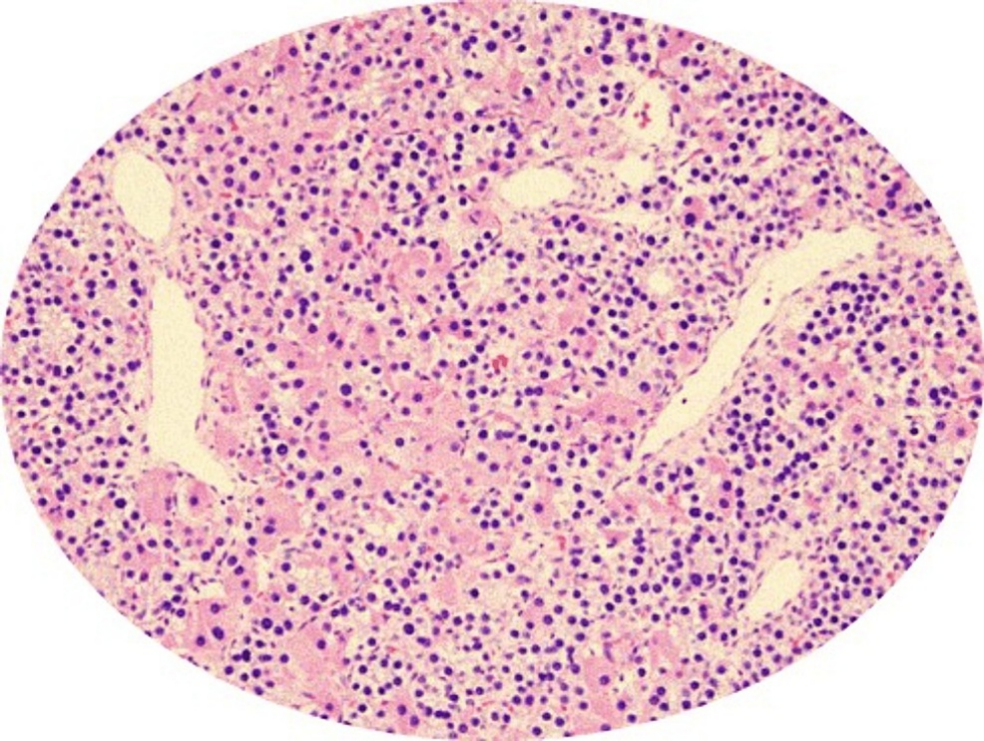
Histopathological examination. Hematoxylin and eosin (H&E) stained parathyroid adenoma. Mainly chief cells are seen.

As the chances of the hungry bone syndrome were high in this case due to the presence of multiple osteolytic lesions, preoperatively patient was given the injection of Zolendronate 5mg intravenously. For her symptomatic hypercalcemia, she underwent unilateral neck exploration and subsequent rigid inferior parathyroidectomy under general anaesthesia. All four parathyroid glands were explored and a 3×2.5×1 cm sized adenoma was excised (Figure 6). Histopathology of the excised gland was consistent with parathyroid adenoma (Figure 7).

After an hour of surgery, her ionised calcium level reduced to below normal range, phosphorus level was high normal, alkaline phosphatase was raised (484 IU/L), and serum intact parathyroid hormone (iPTH) had reduced to less than that of the pre-operative values (Table 5). Parathyroidectomy-induced hypocalcaemia was managed with intravenous five ampules of calcium gluconate in 500ml normal saline (NS) at the rate of 50cc/hour along with oral calcitriol sachets. Her calcium and phosphate levels were closely monitored post-operatively. After eight hours of surgery, serum calcium and serum phosphate levels were subsequently normalised (Table 5).

**Table 5:** Post- Operative serial serum Calcium, ionised Calcium, intact Parathyroid Hormone (iPTH) level and Phosphorus

Investigations	Immediate Post- Operative	Day 1 Post-Operative	On Follow-Up	Normal Value
Total Calcium	7.74	8.9	9.0	8.5-10.5 mg/dl
Ionised Calcium	0.98	1.13	1.15	1.12-1.32 mmol/L
Intact Parathyroid Hormone (PTH)	296	53	47	15-68.3 pg/mL
Phosphorus	4.5	2.54	3.10	2.5-4.5 mg/dL

She was discharged on the tablet calcium 1 gm, vitamin D 1000 units daily and advised adequate hydration along with regular monitoring of serum calcium levels. On regular monthly follow-ups, she was found to be asymptomatic for the last six months with normal serum calcium and iPTH levels (Table 5).

## Discussion

To date, the debate on the relationship between PHPT and pancreatitis has not reached a judgement. However, many studies show the association between these two diseases. Ten studies on the association between PHPT and pancreatitis from different countries were analysed. Eight out of ten studies showed a positive relationship [3] and two studies from India noted the highest rate of pancreatitis (13% and 15%) among patients with PHPT [4,5]. The prevalence of PHPT-associated pancreatitis is only about 1% among all cases of pancreatitis [3]. Patients with PHPT develop a higher rate of pancreatitis than patients without PHPT [6].

Three major mechanisms are proposed in favour of the association between PHPT and pancreatitis: (1) Hypercalcemia resulting from PHPT causes the excessive conversion of trypsinogen into trypsin in the pancreas and trypsin, which is an activated protease, causes autodigestion of the pancreas and finally acute pancreatitis [7]; (2) hypercalcemia promotes the accumulation of calcium within the pancreatic ducts that obstruct them by forming pancreatic calculi which subsequently results in pancreatitis [3]; (3) raised calcium levels along with some genetic mutations like serine protease inhibitors Kazal type 1 (SPINK1) mutation and cystic fibrosis transmembrane conductance regulator (CFTR) mutation greatly increase the risk of pancreatitis in patients with PHPT [8].

The presence of PHPT along with pancreatitis does not change the line of treatment of pancreatitis. Morbidity and mortality of acute pancreatitis is high. Therefore, pancreatitis should be treated first. Once pancreatitis subsides, parathyroidectomy should be performed, as surgery is the only treatment of symptomatic PHPT. A 95% cure rate is reported in PHPT after parathyroidectomy. As parathyroidectomy may result in severe and prolonged hypocalcaemia which is also called 'hungry bone syndrome', after the surgery, the blood levels of calcium and PTH should be regularly monitored [9]. As in our case, the patient may have osteolytic bone lesions without having any significant symptoms, and affected patients should be treated for bone loss.

## Conclusions

Through this case report, we want to highlight the importance of investigating hyperparathyroidism as a possible underlying cause of pancreatitis especially when a patient repeatedly experiences pancreatitis accompanied by elevated levels of calcium in their blood. However, common causes of recurrent pancreatitis like alcohol use, gallstone disease, trauma and hypertriglyceridemia should be ruled out first. The absence of pancreatitis recurrence after parathyroidectomy strengthens the notion of a correlation between hyperparathyroidism and pancreatitis.
